# A Global Characterization and Identification of Multifunctional Enzymes

**DOI:** 10.1371/journal.pone.0038979

**Published:** 2012-06-18

**Authors:** Xian-Ying Cheng, Wei-Juan Huang, Shi-Chang Hu, Hai-Lei Zhang, Hao Wang, Jing-Xian Zhang, Hong-Huang Lin, Yu-Zong Chen, Quan Zou, Zhi-Liang Ji

**Affiliations:** 1 State Key Laboratory of Stress Cell Biology, School of Life Sciences, Xiamen University, Xiamen, Fujian, People's Republic of China; 2 School of Information Science and Technology, Xiamen University, Xiamen, Fujian, People's Republic of China; 3 Department of Pharmacy, National University of Singapore, Singapore, Singapore; 4 The Key Laboratory for Chemical Biology of Fujian Province, Xiamen University, Xiamen, Fujian, People's Republic of China; Université Paris-Sud, France

## Abstract

Multi-functional enzymes are enzymes that perform multiple physiological functions. Characterization and identification of multi-functional enzymes are critical for communication and cooperation between different functions and pathways within a complex cellular system or between cells. In present study, we collected literature-reported 6,799 multi-functional enzymes and systematically characterized them in structural, functional, and evolutionary aspects. It was found that four physiochemical properties, that is, charge, polarizability, hydrophobicity, and solvent accessibility, are important for characterization of multi-functional enzymes. Accordingly, a combinational model of support vector machine and random forest model was constructed, based on which 6,956 potential novel multi-functional enzymes were successfully identified from the ENZYME database. Moreover, it was observed that multi-functional enzymes are non-evenly distributed in species, and that Bacteria have relatively more multi-functional enzymes than Archaebacteria and Eukaryota. Comparative analysis indicated that the multi-functional enzymes experienced a fluctuation of gene gain and loss during the evolution from *S. cerevisiae* to *H. sapiens*. Further pathway analyses indicated that a majority of multi-functional enzymes were well preserved in catalyzing several essential cellular processes, for example, metabolisms of carbohydrates, nucleotides, and amino acids. What’s more, a database of known multi-functional enzymes and a server for novel multi-functional enzyme prediction were also constructed for free access at http://bioinf.xmu.edu.cn/databases/MFEs/index.htm.

## Introduction

In general concepts, multifunctional enzymes (MFEs) are enzymes that play multiple physiological roles. Sometimes, they are further specified as moonlighting enzymes or promiscuous enzymes [1], [2], [3], [4]. Moonlighting enzymes are acknowledged to have at least a single catalytic domain and an additional non-catalytic domain. Both domains execute independent functions, and inactivation of either domain (e.g. by mutation) will not affect another domain [4]. Unlike moonlighting enzymes, promiscuous enzymes are characterized as enzymes of catalytic domains executing several functions, which can be further classified into three subtypes according to mechanisms of enzyme promiscuity: condition promiscuous enzymes, substrate promiscuous enzymes and catalytic promiscuous enzymes. Condition promiscuous enzymes switch their catalytic activities under different reaction conditions, such as various solvent, extreme temperature or altered pH. Substrate promiscuous enzymes are defined as enzymes with relaxed or broad substrate specificity. Catalytic promiscuous enzymes can use the same active site to catalyze different bio-transformations [5]. Normally promiscuous enzymes are annotated with more than one Enzyme Commission (EC) number, however, some promiscuous enzymes have only one given EC number but perform different activities [1].

MFEs are beneficial to living systems by providing competitive survival edges in a variety of ways. They are able to employ alternative approaches to coordinate multiple activities and regulate their own expression [2], which demonstrates an evolutionary advantage as part of a clever strategy for generating complexity from existing proteins without expansion of genome [6], [7], [8]. Moreover, combination of multiple functions enables an enzyme to act as a switch point in biochemical or signaling pathways so that a cell can rapidly respond to changes in surrounding environment [9]. Multi-functionality seems to be a common mechanism of communication and cooperation between different functions and pathways within a complex cellular system or between cells [3].

In recent years, more and more novel multifunctional enzymes are being discovered. Identification of MFEs and subsequent investigation of their mechanistic and structural basis of multi-functionality become an shortcut important for studying biological roles of enzymes, their multiple activities in protein engineering [10] and inhibitor design [11] . As a complementary solution to experimental methods, current sequence analysis algorithms (alignment, clustering and motif approaches) have shown their distinct capabilities in disclosing individual functions of MFEs [12]. Algorithms based on remote homology, e.g. PSI-BLAST (Position Specific Iterative-Basic Local Alignment Search Tool) [13] , have been found to give good performance in finding alternative functions of MFEs [12]. However, in some cases, it is difficult to determine whether the predicted multiple functions by these methods are due to true multi-functionality or false identification [3], [7], [14]. It is acknowledged that active sites of MFEs with multiple catalytic activities are inherently reactive environments packed with nucleophiles, electrophiles, acids, bases and cofactors. Sometimes, common structural and physicochemical features are presented when MFEs execute similar functions regardless of their high diversities in sequence. Therefore, proper characterization of these features will be helpful for mechanistic understanding of enzyme multi-functionality, and furthermore can provide clues to characterize novel MFEs when they can’t be properly identified by homology-based approaches.

## Materials and Methods

### Search of MFEs and Classification

In this study, a keyword search of “multifunctional enzyme” against the UniProt Knowledgebase (UniProtKB, release-2011-08) [15] was demonstrated to maximally collect MFEs. This was followed by manual validation that each MFE performs at least two distinct physiological functions, including one catalytic activity and one or more additional catalytic/regulatory/binding actives. Finally, a total of 6,799 MFEs were collected and validated. These MFEs cover typical moonlighting enzymes, promiscuous enzymes and MFEs that are difficult to be classified into above two groups. According to the number of functional domains (Pfam domain) in protein, they were further divided into two classes: 1,235 MFEs with single multi-activity domain (SMAD-MFEs) and 5,564 MFEs with multiple catalytic/functional domains (MCD-MFEs) respectively. Roughly, many SMAD-MFEs are promiscuous enzymes and many MCD-MFEs are moonlighting enzymes. Such classification would be helpful for later characterization and discovery of MFEs.

### Identification of MFEs

#### Dataset preparation

A total of 6,782 known MFEs whose amino acids length are more than 100 were chosen as positive dataset for model construction. The non-MFE proteins (negative data) were selected from seeds in the Pfam database [16] as following: Each Pfam protein family represents a cluster of proteins with similar domain architecture. The negative protein families were achieved by excluding those Pfam domain families that contain at least one MFE member, so that all proteins that have similar domain architecture as known MFEs were maximally removed. The negative dataset were then generated by randomly picked up one protein seed (amino acids length are more than 100 as well) from these negative Pfam protein families. In this way, the coverage (different domain architectures) of negative dataset was enhanced and, at the same time, the possible bias in negative data selection was reduced to the most extent. Finally, 10,714 non-MFE proteins were assigned into the negative data pool.

To be eligible for model construction, every protein sequence was represented by specific feature vector assembled from encoded representations of nine tabulated residue properties including amino acid composition, hydrophobicity, normalized Van der Waals volume, polarity, polarizability, charge, surface tension, secondary structure and solvent accessibility for each residue in the sequence. Three descriptors, composition, transition and distribution, were used to describe global composition of each property. Composition is the number of amino acids of a particular property (such as hydrophobicity) divided by the total number of amino acids. Transition characterizes the percent frequency with which amino acids of a particular property is followed by amino acids of a different property. Distribution measures the chain length within which the first, 25, 50, 75 and 100% of the amino acids of a particular property is located respectively. All descriptors for each property were computed and combined to form the feature vector as described in previous literatures [17]. Finally, a feature vector of 188 elements was generated to represent a protein sequence.

#### Construction of SVM model

Support vector machine (SVM) is based on the structural risk minimization principle of statistical learning theory. The detailed methodology of the SVM training and classification has been well described in the literature [18], [19]. In principle, the proteins, represented as feature vectors, were mapped into a multi-dimensional (here, 188 dimensions) feature space. A hypothetical hyper plane was used to classify these proteins into one of two classes: MFEs (the positive class) or non-MFE proteins (the negative class). This hyper plane was determined by finding a vector **w** and a parameter *b* that minimized 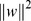
to satisfy the following conditions: 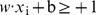,for 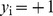 (positive class) and 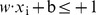,for 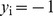 (negative class). Here **x**_i_ is a feature vector, **y**_i_ is the class index, **w** is a vector normal to the hyper plane, and 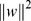 is the Euclidean norm of **w**. In this study, we adopted the build-in libsvm algorithm in the WEKA program for model construction.

#### Construction of RF model

Random forest (RF) is a classifying algorithm of ensemble learning. It is called as “forest” because it consists of several decision trees. The algorithm has been properly described in previous application [20]. There are two major ideas of RF, bagging and random feature selection. In bagging, classifiers are trained on a bootstrap training data and the prediction is voted by these classifiers. RF selects some features randomly and splits them at each node when constructing decision trees. Each tree in the forest is constructed to the largest extent possible without any pruning. This procedure will be iterated over all trees in the ensemble, and the average vote of all trees is reported as RF prediction. In this study, we adopted the embedded RF algorithm in the WEKA program for prediction.

#### Evaluation of model

As a discriminative method, the performances of SVM classification and RF classification were measured by the quantity of true positive TP, false negative FN, true negative TN, and false positive FP. In addition, the specificity SP =  TN/(TN+FP), the sensitivity SE =  TP/(TP+FN), the positive prediction value PPV  =  TP/ (TP + FP) and the overall prediction accuracy P  =  (TP + TN)/ (TP+FN+TN+FP) were also evaluated.

## Results and Discussion

### Sequential and Structural Preference of MFEs

#### Physiochemical propensities

In most cases, sequence conservation can properly explain similar functions of different enzymes. However, exceptions were reported that some functional groups are un-conserved in sequence composition but mediate same enzymatic mechanistic role due to their structural flexibility at the active site [21]. The structural flexibility however still maintained the similar conformation changes at the active site so that these functional groups were able to execute same enzymatic function. It seems that such functional plasticity may not be sufficiently described by commonly used homology-based approaches. Therefore, recognition of structural and physicochemical features that can properly describe this plasticity may be helpful for identification of MFEs by non-homology-based methods like SVM and RF. In this work, total of nine feature properties were used to describe structural and physicochemical characteristics of each protein. These properties have been routinely used for classification of proteins of different structural and functional classes [17], [19], [22]. It was acknowledged that not all these features contribute equally to protein classification; some have been found to play relatively more prominent role than others [22]. It is thus of interest to examine which feature properties are dominant in classification of MFEs.

Previously, contribution of individual feature property to protein classification was investigated [22]. Similar approach was also employed in present study. It was found that the charge, polarizability, hydrophobicity, and solvent accessibility play more prominent role than other feature properties. This is agreed with previous studies that some MFEs, e.g. ADP-ribosyl cyclase and CD38, can switch functions at different pH, indicating the importance of polarity, charge distribution and solvent accessibility in determining their multi-functionality [23]. Multiple protein-interacting modules of some MFEs, e.g. High-voltage-activated Ca2+ channels, involve in hydrophobic interactions [24]. Some MFEs, e.g. neuronal nitric oxide synthase, have large solvent-exposed hydrophobic surface that contains a cavity rimmed with charges [25]. These sequential features are useful to identify novel MFEs.

#### Identification of novel MFEs

Identification of novel MFEs may be one of the best ways in understanding multiple functionalities of enzymes. In present study, a combinational model of support vector machine and a random RF model was trained and optimized as described in the methodology section. According to our previous analyses on the physiochemical and structural preference of known MFEs, nine sequential and structural features were adopted. These two models were optimized by five-fold cross validation and the performances were given in **Table 1**.

**Table 1:** The performances of SVM model and RF model in classification of MFEs.

	Positives	Negatives	TP	FP	TN	FN	SP (%)	SE (%)	PPV (%)	Q (%)
**SVM**	6,782	10,714	5,642	1,435	9,279	1,140	86.6	83.2	79.7	85.3
**RF**	6,782	10,714	6,368	632	10,082	414	94.1	93.9	91.0	94.0

The prediction were evaluated by parameters of TP (true positive), FN (false negative), TN (true negative), FP (false positive), specificity SP =  TN/(TN+FP), sensitivity SE =  TP/(TP+FN), positive prediction value PPV = TP/ (TP+FP) and overall accuracy Q =  (TP+TN)/ (TP+FN+TN+FP).

The optimized models were then applied to screen the ENZYME database [26] for identification of novel MFEs. A probability value ranging from 0 to 1.0 (or 0 to 100%) was given to evaluate each model prediction. A value close to 100% indicates the higher possibility of prediction. Satisfying both SVM model (probability >90%) and RF model (probability >80%), totally 6,956 novel MFEs and 6,071 known MFEs were identified with from 205,173 enzymes (amino acids length are more than 100) in the ENZYME database (Release of 21-Mar-12). Among the 6,782 currently known MFEs collected from UniProt knowledgebase, 6,071 were successfully identified from the ENZYME database, 50 were excluded because of low prediction probability, and 661 haven’t been recorded by the ENZYME database yet but annotated in the UniProtKB. The complete list of both known and predicted MFEs can be acquired from a novel MFE database at http://bioinf.xmu.edu.cn/databases/MFEs/index.htm.

The database was curated on Red Hat Linux release 9 operating system. The data were managed by the RDBMS Oracle *10* *g*. Interactive user interfaces and search engines were coded by PHP and JavaScript. Three methods were developed for rapid access of the MFEs database. They are briefly described as follows: The database offers a quick search method to retrieve information via keyword query forms. To initiate a search, user is required to type a partial or full keyword in the text field of query form. Wild-card characters like "*, &, ?" are not supported. Once a query is submitted, a list of protein names that meet the query criteria will be responded in alphabet order respectively. Clicking on a protein will lead to the detailed information page, where the detailed information of enzyme is presented in three sections of General Information, Features and MFEs Type. Besides, an ID search method is available for accurate access of database by just providing a UniProtKB AC, EC number, or Pfam ID. The database also offers an alternative browse method for direct retrieval of MFE information by selecting an enzyme from the species list, EC number list or enzyme name list.

Additionally, an on-line classification system for novel MFEs was also constructed for public access http://jing.cz3.nus.edu.sg/cgi-bin/sime.cgi. The prediction is based on the pre-established and refined machine learning models of SVM, RF or their combination. Combination of these two different algorithms, to a large extent, reduces the false positives. However, several factors may more or less affect its performance. One is the diversity of protein samples used for developing classification systems. It is likely that not all possible types of MFEs and non-MFEs were adequately represented in the training set. This can be improved with the availability of more diverse protein sequences and improved knowledge about MFEs. A broad spectrum of MFEs of diverse functions may also affect the performance of our SVM and RF models to some extent.

#### Structural preference

**Figure 1:**
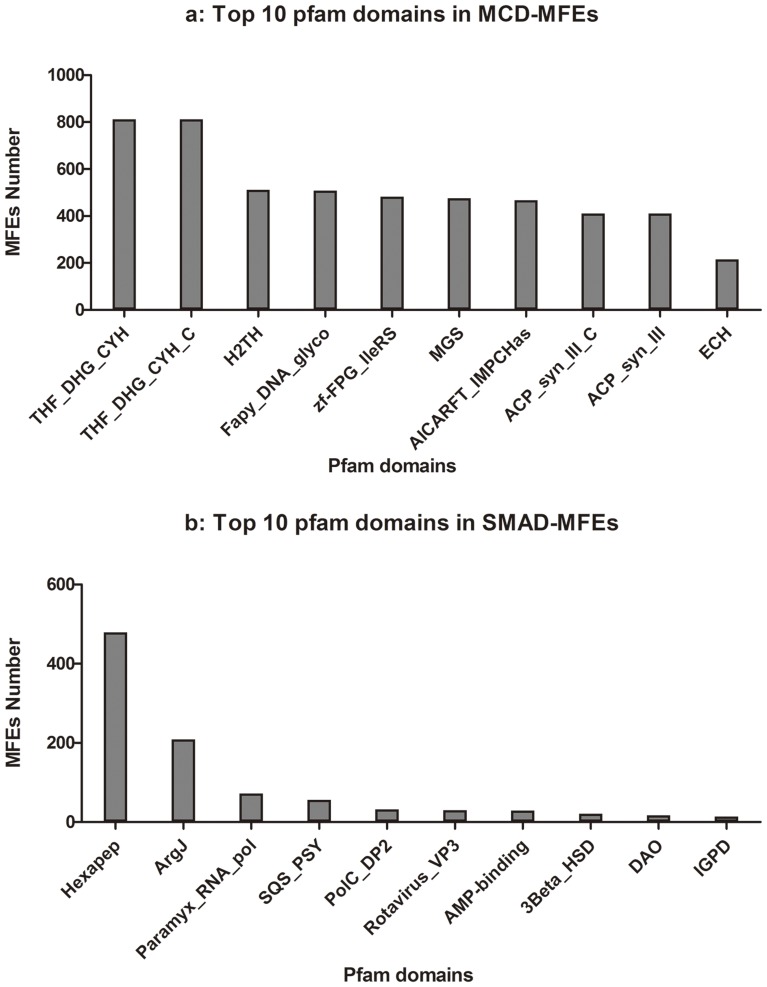
The top 10 frequently used Pfam domain families for known MFEs. It is noted that about 17% of SMAD-MFEs contain ArgJ. It plays key role in both N-acetylglutamate synthase and ornithine acetyltransferase activities in the cyclic version of arginine biosynthesis.

**Figure 2:**
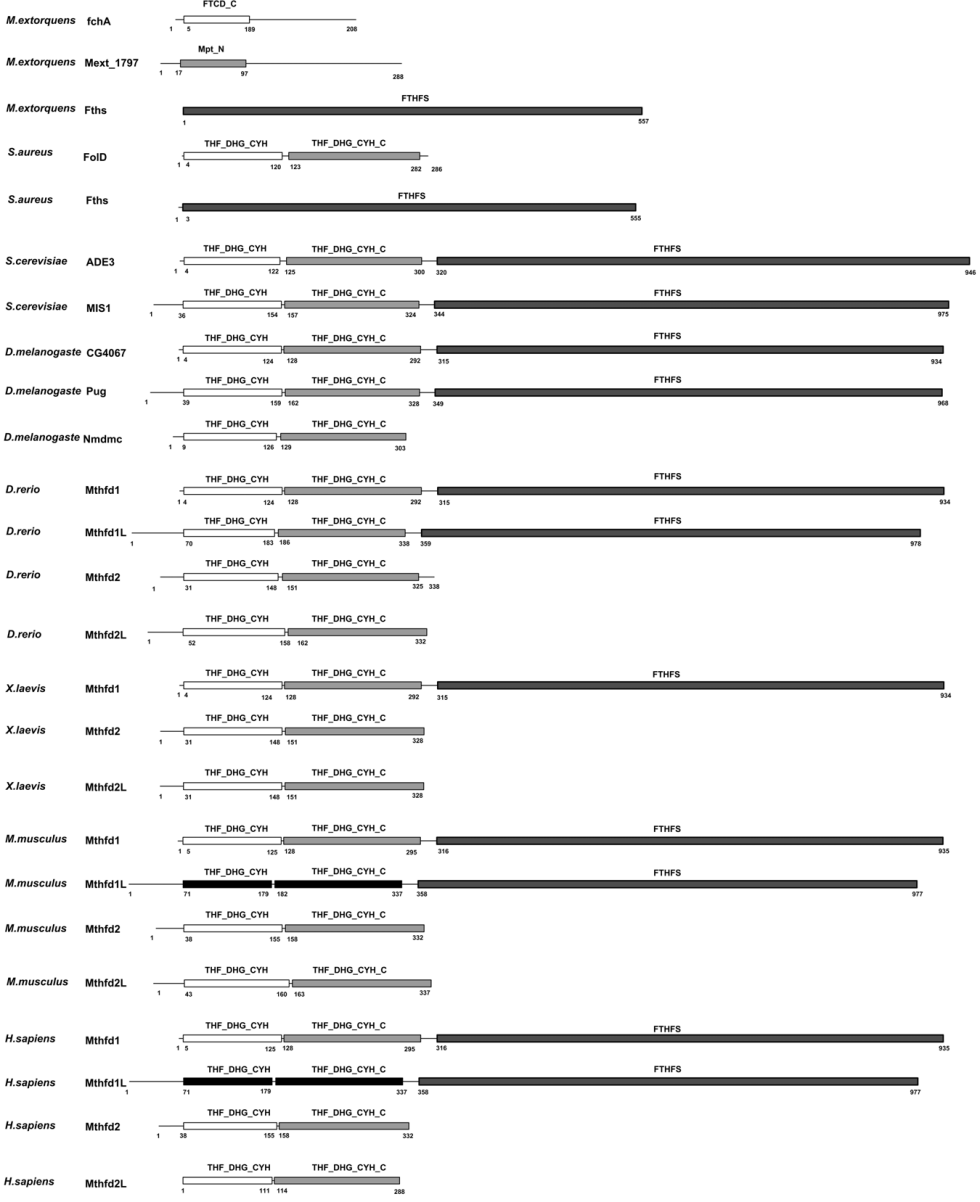
The evolution path of C-1-tetrahydrofolate synthase in eukaryotic representatives including *M. extorquens*, *S. aureus*, *S. cerevisiae*, *D. melanogaster*, *D. rerio*, *X. laevis*, *M. musculus*, *H. sapiens*. It illustrated how three independent proteins (domains) fused and mutated during the evolutionary path, which resulted in the gain and loss of multiple-functionality. The THF_DHG_CYH and THF_DHG_CYH_C domains of human and mouse Mthfd1L proteins illustrated in dark block of net pattern were mutated and lost tetrahydrofolate dehydrogenase/cyclohydrolase activities.

Knowledge of domain composition provides valuable insights into the mechanism of MFEs. The top 10 Pfam domains in two classes of MFEs were listed in **Figure 1** **a & b** respectively. One of the most frequent domain in SMAD-MFEs (**Figure 1b**) is ArgJ (Pfam ID: PF01960), which plays key role in both N-acetylglutamate synthase (EC 2.3.1.1) and ornithine acetyltransferase (EC 2.3.1.35) activities in the cyclic version of arginine biosynthesis [27]. Structural analysis of ArgJ domain indicates that its complete active-site is defined by some disconnected residues, potentially the protein C-terminus. The coming out and going in movement of C-terminus at the active site likely enables ArgJ to execute two different substrates-specific bindings [28]. The flexibility of structure at the active sites might be a common mechanism for SMAD-MFEs perform their multi-functionality. Just like some scaffold proteins having intrinsic disorder regions, SMAD-MFEs may change their conformations under different conditions, thus play different physiological roles. For example, a SMAD-MFE, human apurinic/apyrimidinic endonuclease (APE), switches its role of either base excision or nucleotide incision repair by conformational changing of substrate binding domain before the chemical cleavage step [29]. Unlike the SMAD-MFEs, MCD-MFEs realize their multi-functionality via domain combination. It is interesting that some of the frequently-used domains of MCD-MFEs appear in pairs. For instance, a number of eukaryotes enzymes contain both tetrahydrofolate dehydrogenase/cyclohydrolase NAD(P)-binding domain, (THF_DHG_CYH_C, Pfam ID: PF02882) and catalytic domain, (THF_DHG_CYH, Pfam ID: PF00763), which present separately in many prokaryotes as single-function enzymes. This might be the clues of gene fusion in the process of protein specificity (**Figure 2**).

**Figure 3:**
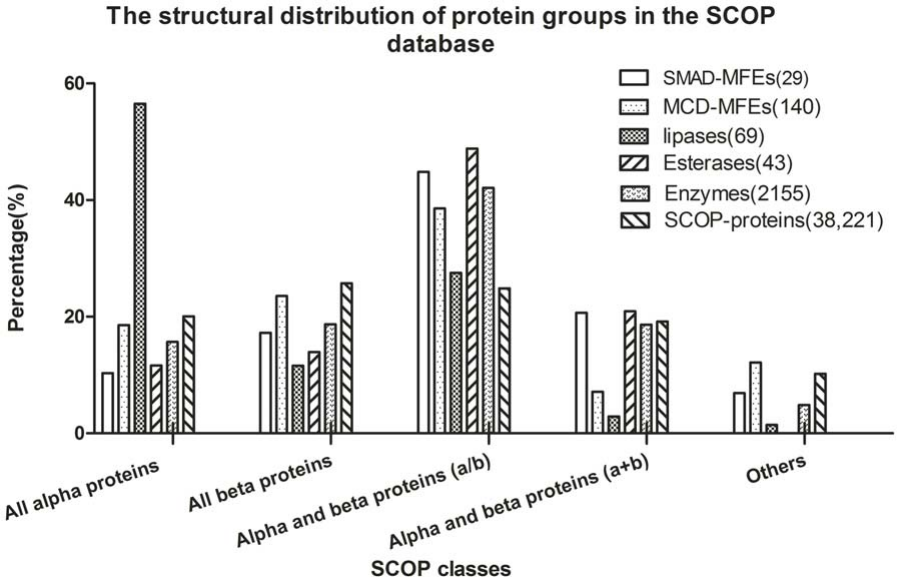
The structural distribution of protein groups in the SCOP database. It is noted that about 38.57% of MCD-MFEs, 44.83% of SMAD-MFEs, 48.84% of esterases and 42.09% of enzymes belong to alpha and beta proteins (a/b); comparatively, only 24.85% of SCOP proteins belong to a/b topology. In this analysis, 140 known MCD-MFEs, 29 known SMAD-MFEs, 69 lipases, 43 esterases, 2155 enzymes, and 38,221 proteins were included.

To have an overview of MFEs’ structural propensities, the distribution of several protein groups in Structural Classification of Proteins (SCOP) database [30] was investigated. The analysis covers 140 known MCD-MFEs, 29 known SMAD-MFEs, 2,155 enzymes and total 38,221 Protein Data Bank (PDB) Entries included in the SCOP 1.75 release database (June 2009). As illustrated in **Figure 3**, about 38.57% of MCD-MFEs and 44.83% of SMAD-MFEs belong to alpha and beta proteins (a/b); while only about 24.85% of total proteins in SCOP database are in a/b topology. It seems that MFEs have a structural propensity in alpha and beta topology. The propensity of a/b topology would be a general characteristic of enzyme.. Be aware that these results were achieved subject to current availability of protein structures in SCOP, which is limited and bias due to the difficulty in structure determination. However, some recent studies proposed that alpha and beta topology was common for moonlighting proteins [31], [32], which would be a good case to support our finding.

### Physiological Roles of MFEs

Biological pathways are networks of molecular interactions, which provide valuable information of complex cellular reactions in molecular level. Herein, the physiological roles of MFEs were investigated via searching against Kyoto Encyclopedia of Genes and Genomes (KEGG) database [33]. Among the 4,935 currently known MFEs with KEGG Ontology (KO) annotation and pathway information, about 91.31% of total MCD-MFEs and 96.31% of total SMAD-MFEs were involved in one or two distinct cellular processes (**Table 2**).

**Table 2:** The statistics of MFEs by number of KEGG biological pathways they are involved in.

Num# of Pathways	MCD-MFEs		SMAD-MFEs		MFEs of Archaea		MFEs of Bacteria		MFEs of Eukaryota	
	Num^#^	PCT^*^(%)	Num^#^	PCT^*^(%)	Num^#^	PCT^*^(%)	Num^#^	PCT^*^(%)	Num^#^	PCT^*^(%)
1	2,377	57.65	729	89.78	24	22.02	2,764	63.64	317	65.63
2	1,388	33.66	53	6.53	78	71.56	1,277	29.40	86	17.81
3	89	2.16	7	0.86	0	0	56	1.29	40	8.28
4	37	0.90	13	1.60	0	0	39	0.90	11	2.28
5 and more	232	5.63	10	1.23	7	6.42	206	4.74	29	6.00

Num^#^: Number; PCT^*^: Percentage.

Totally, 4,123 known MCD-MFEs and 812 known SMAD-MFEs were included in this statistics, covering 109, 4,343, and 483 known MFEs were respectively in Archaea, Bacteria and Eukaryota respectively.

According to KO annotation, the MCD-MFEs participate in 6 level one, 35 level two, and 140 level three pathways; while SMAD-MFEs were involved in 6 level one, 29 level two and 92 level three pathways. The distributions were illustrated in **Figure S1 & S2** respectively. It looks that majority of MFEs (81.2% and 97.2% for MCD-MFEs and SMAD-MFEs respectively) were involved in metabolism pathways, over 80% of which were carbohydrate metabolism (CAR, KEGG: map01110), lipid metabolism (LIP, KEGG: map01130), nucleotide metabolism (NUC, KEGG: map01140), amino acid metabolism (AAC, KEGG: map01150) and metabolism of cofactors and vitamins (COF, KEGG: map01190). Moreover, about 50% of MCD-MFEs were involved in pathways of CAR and COF, which can be, to some extent, explained by a large number of the tetrahydrofolate dehydrogenase/cyclohydrolase family members. Considering the very conservation of metabolic enzyme in three life domains [34], the enrichment of MFEs in several metabolism processes indicates that they could be the early enzymes, and their multi-functionality could be an efficient solution for early life forms to preserve as many basic metabolic activities as possible in small genome size. This inference is agreed with a recent study that promiscuous enzymes are mainly involved in amino acid and lipid metabolisms, which might be associated with the earliest form of biochemical reactions [1].

**Table 3:** The distribution of MFEs in four life domains of Archaea, Bacteria, Virus and Eukaryota.

Domains	Organism Num^#^		Enzyme Num^#^		Average enzyme Num^#^ in each organism	
	MCD-MFEs	SMAD-MFEs	MCD-MFEs	SMAD-MFEs	MCD-MFEs	SMAD-MFEs
**Archaea**	40	36	71	66	1.78 (±0.81)	1.83 (±1.81)
**Bacteria**	590	380	4413	754	7.48 (±6.18)	1.98 (±1.21)
**Eukaryota**	143	120	633	270	4.43 (±5.00)	2.25 (±1.74)
**Virus**	156	77	446	145	2.86 (±2.50)	1.88 (±1.26)

Num^#^: Number.

Totally, 5,554 known MFEs of multiple catalytic/functional domains (MCD-MFEs) and 1,274 known MFEs of single multi-activity domain (SMAD-MFEs) were included in the statistics. It was noted bacteria are superior in both total number and average number of known MCD-MFEs and SMAD-MFEs than other three domains.

**Table 4:** The statistics of known MFEs in seven eukaryotic model organisms.

Organisms	MCD-MFEs Num#	SMAD-MFEs Num#	Total MFEs Num#	Total Enzymes Num# *	PCT# (%)
***S. cerevisiae***	90	15	105	1,568	6.70
***C. elegans***	11	0	11	661	1.66
***D. melanogaster***	13	1	14	607	2.31
***D. rerio***	3	1	4	372	1.08
***X. laevis***	6	0	6	477	1.26
***M. musculus***	59	13	72	2,789	2.58
***H. sapiens***	83	22	105	2,795	3.76

* : Currently known enzymes in the ENZYME database.

# Num: Number; PCT: Percentage.

It showed that the MFEs experienced a fluctuation of MFE gain and loss in 7 eukaryotic model organisms including *S. cerevisiae*, *C. elegans*, *D. melanogaster*, *D. rerio*, *X. laevis*, *M. musculus*, *H. sapiens*. The average number of MFEs decreased from *S. cerevisiae* to *D. rerio*, and then increased from *X. laevis* to mammal animals.

### Gain and Loss of Multiple Functionalities

According to our analyses, bacteria have more MFEs than archaebacteria and eukaryotes in both total and average content of MFEs (**Table 3**). This result was achieved under the circumstance that, relatively, bacteria were more studied than archaebacteria and eukaryotes. It is also noticed that the content of MFEs in bacteria are very unbalanced. Some bacterial organisms have many MFEs, while some have few. Similar unbalance was also observed in lower eukaryotes. In this study, a close statistics of known MFEs in seven representative eukaryotic model organisms was demonstrated as well, including *S. cerevisiae*, *C. elegans*, *D. melanogaster*, *D. rerio*, *X. laevis*, *M. musculus*, *H. sapiens*. They were roughly arranged and compared in an ascent evolutionary order according to their first appearance in geological time. It showed that the MFEs experienced a fluctuation of enzyme gain and loss: decrease from *S. cerevisiae* to *D. rerio* and then increase from *X. laevis* to *H. sapiens* (**Table 4**). For early simple life forms (e.g. *S. cerevisiae*), comparatively small genome limited their protein-coding capacity. As an alternative solution, ancient enzymes have to broaden their substrate specificity or adopt multiple functions, which may be achieved by gene duplication in tandem accompanying with mutational modifications [6]. With the emergence of multi-cell eukaryotic organisms, complex intra-cellular and inter-cellular interactions required more accurate and diverse enzymatic activities. On one hand, multi-functional enzymes might be specialized so as to execute a definite catalytic function. For instance, an early multifunctional enzyme catalyzing consecutive steps might diversify into two more specific and efficient enzymes today, each of which catalyzes only one step in the pathway [35]. On the other hand, novel multi-functional enzymes emerged when broader substrates and reaction specificities are subsequently captured by adaptive evolution [36]. For example, the last two steps of de novo biosynthesis of CoA are catalyzed by two independent enzymes, phosphopantetheine adenylyltransferase (EC 2.7.7.3; PPAT) and dephosphocoenzyme A kinase (EC 2.7.1.24; DPCK), in bacteria and before metazoan, however, these two steps are now accomplished by a bifunctional CoA synthetase containing both PPAT and DPCK domains in metazoan [37].

The multiple functionalities of MFEs were inherited in most cases during species evolutionary. Several rounds of genome duplication during species evolution expanded the gene number in an explosive manner, which enabled the rapid specification of MFEs by generating paralogs. Some of these MFE paralogs lost part of or even all (the pseudo-gene) their functions by means of gene mutation, alternative splicing, nonsynonymous substitution, exon recombination and etc. A typical example is the tetrahydrofolate dehydrogenase/cyclohydrolase family. Most tetrahydrofolate dehydrogenase/cyclohydrolase family members (768 out of all 1,180 species except viruses) are well conserved in possessing both methenyltetrahydrofolate cyclohydrolase and methylenetetrahydrofolate dehydrogenase activities. In Eukaryota, these two activities usually present together. As shown in **Figure 2**, four Mthfd paralogs contain the THF_DHG_CYH and THF_DHG_CYH_C domains. They all perform these two activities except human mitochondrial monofunctional C1-tetrahydrofolate (C1-THF) synthase encoded by gene MTHFD1L. The human mitochondrial C1-THF synthase is 61% identical to its human cytoplasmic isozyme Mthfd1, however, lacks amino acids that are critical for the binding of NADP+ and folate [38], [39]. The loss of multiple functionalities of MFEs in some species may suggest a potential mechanism of novel protein generation or functional regulation of biological pathways.

On the other side, interacting proteins (direct interaction or upstream-downstream proteins in a pathway) however integrated their functions to achieve more effective cell device via mechanisms like gene fusion. In this study, we compared the domain structures of 25 enzymes containing either of methylenetetrahydrofolate dehydrogenase, methenyltetrahydrofolate cyclohydrolase or formyltetrahydrofolate synthetase from eight representative organisms including two prokaryotes and six eukaryotes (**Figure 2**). It was observed that, in most cases, Mthfd enzyme families gained their multiple functions in a way of gene fusion. In prokaryotes, e.g. *M. extorquens*, methylenetetrahydrofolate dehydrogenase activity, methenyltetrahydrofolate cyclohydrolase activity and formyltetrahydrofolate synthetase activity are realized by three independent monofunctional proteins, except for a bifunctional cyclohydrolase/dehydrogenase in *E. coli*, *C. thermoaceticum*, and *etc.* However, in eukaryotes, these three activities are normally executed by a single trifunctional protein, C1-THF synthase [40]. Similar phenomena can be observed in the multifunctional protein 17b-HSD4 [41].

### Conclusion

In present study, we globally analyzed MFEs on different aspects of structure, function and evolution. Some common patterns of MFEs were identified, and for the first time, a combinational model of SVM and RF was constructed for novel MFE prediction. It is noticed that many results presented in this study were achieved in basis of current availability of MFEs, which were affected by bias of data availability. For this reason, some conclusions might not be well agreed with previous findings which were also inferred from current knowledge of MFEs. Nevertheless, our findings will to some extent help systematic understanding of MFEs and their roles in crosstalk between various cellular processes.

## Supporting Information

Figure S1 **The KEGG ontology analysis of known MCD-MFEs.** Total 4,123 known multifunctional enzymes of multiple catalytic/functional domains (MCD-MFEs) were included in the analysis.
(TIF)

Figure S2 **The KEGG ontology analysis of known SMAD-MFEs.** Total 812 known multifunctional enzymes of single multi-activity domain (SMAD-MFEs) were included in the analysis.
(TIF)
