# Supplementary figures and images for: A Global Characterization and Identification of Multifunctional Enzymes
Source: PLoS One. 2012 Jun 18;7(6):e38979. doi: 10.1371/journal.pone.0038979 (PMC3377604; doi:10.1371/journal.pone.0038979)

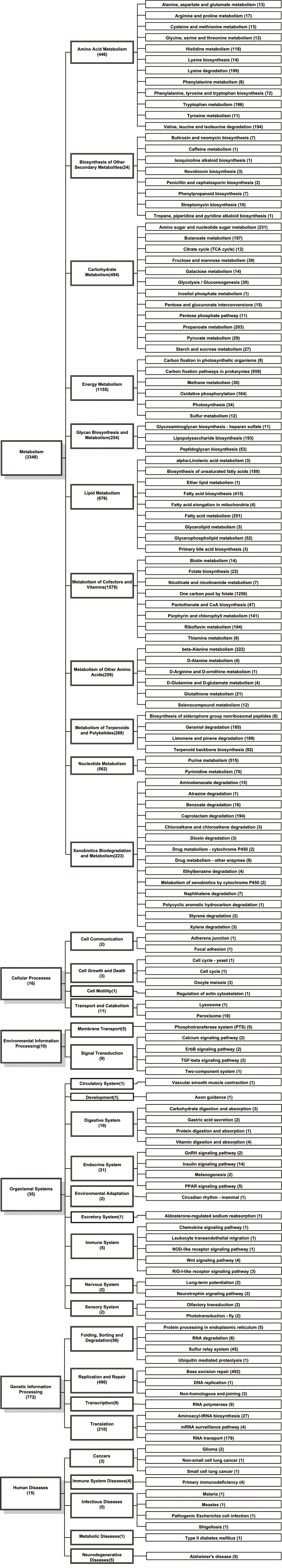

Supplement: Figure S1 — The KEGG ontology analysis of known MCD-MFEs. Total 4,123 known multifunctional enzymes of multiple catalytic/functional domains (MCD-MFEs) were included in the analysis. (TIF) [file pone.0038979.s001.tif]

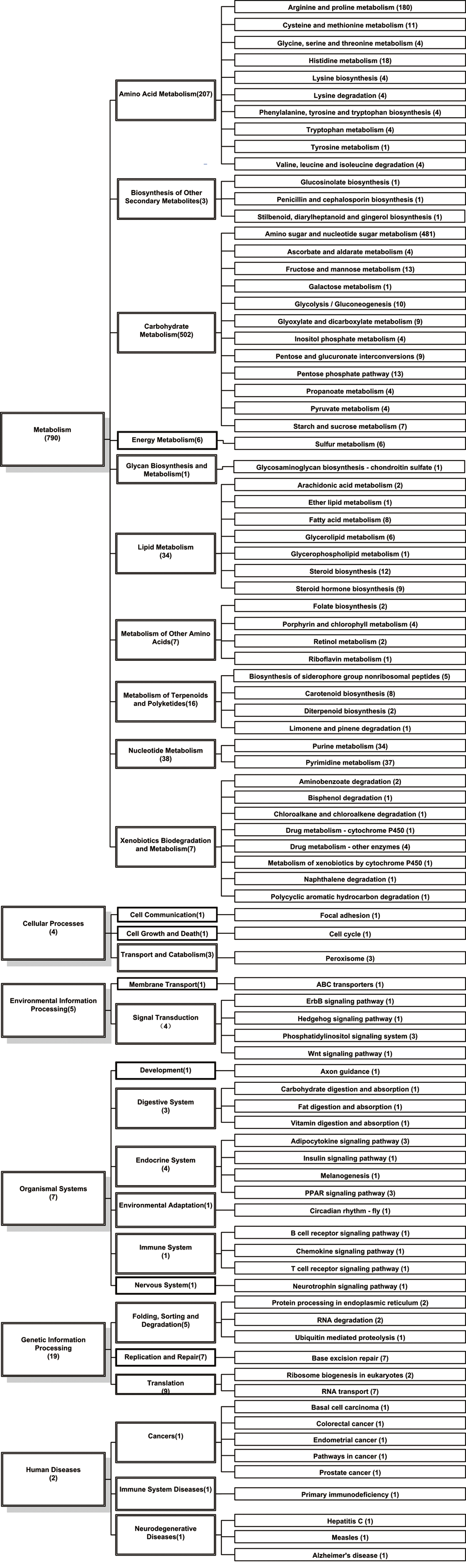

Supplement: Figure S2 — The KEGG ontology analysis of known SMAD-MFEs. Total 812 known multifunctional enzymes of single multi-activity domain (SMAD-MFEs) were included in the analysis. (TIF) [file pone.0038979.s002.tif]
